# Trends in cell medicine: from autologous cells to allogeneic universal-use cells for adoptive T-cell therapies

**DOI:** 10.1093/intimm/dxad051

**Published:** 2024-01-08

**Authors:** Hiroshi Kawamoto, Kyoko Masuda

**Affiliations:** Laboratory of Immunology, Institute for Life and Medical Sciences, Kyoto University, Kyoto 606-8507, Japan; Laboratory of Regenerative Immunology, International Center for Cell and Gene Therapy, Fujita Health University, Toyoake 470-1192, Japan; Laboratory of Immunology, Institute for Life and Medical Sciences, Kyoto University, Kyoto 606-8507, Japan

**Keywords:** adoptive cell therapy, CAR-T cell therapy, HLA, iPS cells, NK cells

## Abstract

In currently ongoing adoptive T-cell therapies, T cells collected from patients are given back to them after *ex vivo* activation and expansion. In some cases, T cells are transduced with chimeric antigen receptor (CAR) or T-cell receptor (TCR) genes during the *ex vivo* culture period in order to endow T cells with the desired antigen specificity. Although such strategies are effective in some types of cancer, there remain issues to be solved: (i) the limited number of cells, (ii) it is time-consuming, (iii) it is costly, and (iv) the quality can be unstable. Points (ii) and (iv) can be solved by preparing allogeneic T cells and cryopreserving them in advance and methods are being developed using healthy donor-derived T cells or pluripotent stem cells as materials. Whereas it is difficult to solve (i) and (iii) in the former case, all the issues can be cleared in the latter case. However, in either case, a new problem arises: rejection by the patient’s immune system. Deletion of human leukocyte antigen (HLA) avoids rejection by recipient T cells, but causes rejection by NK cells, which can recognize loss of HLA class I. Various countermeasures have been developed, but no definitive solution is yet available. Therefore, further research and development are necessary.

## Teaser

Cell medicine: from autologous to allogeneic cells

## Introduction

**Figure 1.**
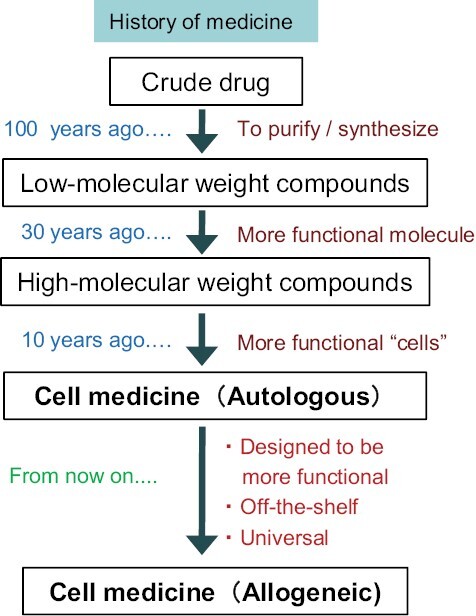
The history of drug development. For a long time in the history of mankind, the era of crude drugs continued. In the 20th century, methods for purifying components from natural products were established. About 30 years ago, proteins such as antibodies and cytokines began to appear in the main products of pharmaceutical companies. Triggered by CAR-T cell therapy, which appeared in the 2010s, major pharmaceutical companies have entered the autologous cell medicine field one after another. In the near future, there will be a shift from the strategy of using autologous cells to the strategy of using allogeneic cells that have been endowed with some additional functions in advance as a universal, off-the-shelf cell medicine.

Here we take a bird’s-eye view of cell therapy from the perspective of the history of drug development (Fig. 1). For a long time in the history of mankind, the era of “crude drugs” continued. It was not until the 20th century that methods for isolating and purifying active ingredients were established. Eventually, they came to be synthesized and, from then on, low-molecular-weight compounds became the centerpiece of drug discovery. On the other hand, since proteins such as antibodies and cytokines need to be produced by cells, many pharmaceutical companies do not consider them to be one of the main pillars of drug discovery. However, many companies now have antibody preparations as their main products.

Antibodies and cytokines have specific physiological activities and can perform tasks that are difficult for low-molecular compounds. In this context, cells possess properties not achieved by proteins, such as strong cytotoxicity, migration, and proliferation. Indeed, chimeric antigen receptor (CAR)-T cell therapy, which confers antibody specificity to “autologous” T cells, has shown remarkable efficacy against certain hematological cancers, and since its emergence in the 2010s, major pharmaceutical companies have embraced this strategy one after another.

Along with this trend, it is certain that the era of cell medicine using “allogeneic” cells, which enables high versatility and immediate delivery, will come next. However, in allogeneic settings, the problem of “immune rejection” may arise. In this article, we will firstly list issues in an autologous setting and explain how to address these issues by using allogeneic cells, and then discuss the new issues of immune rejection that then occur.

## Autologous cells to allogeneic cells

**Figure 2.**
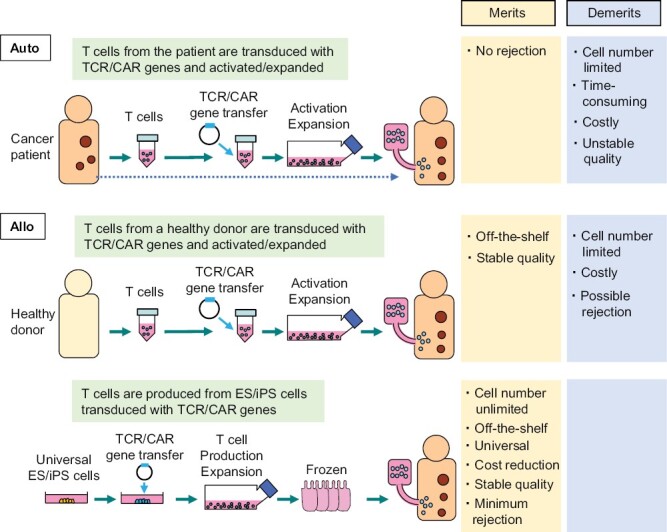
Issues of cell therapy using autologous T cells and solutions offered by allogeneic T cells. Current autologous T cell therapy has issues such as (i) the number of cells that can be produced is limited, (ii) it takes time, (iii) it is costly, and (iv) the quality is unstable because it depends on the quality of the patient’s T cells. When allogeneic T cells collected from healthy donors are used as materials, (ii) and (iv) can be resolved, but (i) and (iii) remain to be solved. By using highly versatile ES and iPS cells as materials, it is possible to solve all of the issues (i)–(iv).

The current CAR-T cell therapy and T-cell receptor (TCR) T-cell therapy use autologous T cells as materials. Regarding T cells, whereas cytotoxic T lymphocytes (CTLs) play the main roles in killing tumor cells, helper T cells are thought to work to enhance the effect of CTLs, and thus both are used in a mixed manner. Although this method has been shown to be effective, there remain some issues. Since autologous T cells are used as materials, (i) the number of cells that can be produced is limited, (ii) production takes time, (iii) production costs are high, and (iv) the quality of the product is unstable because high-quality T cells cannot always be obtained from the patient (Fig. 2, upper lane).

In addition to these issues, adverse effects caused by preconditioning for lymphodepletion could also be listed. Preconditioning is often performed in both autologous CAR-T and TCR-T cell therapies to induce the expansion of transferred T cells, using fludarabine or cyclophosphamide. On the other hand, preconditioning may exert some adverse effects such as increased risk of infection and specific toxicity caused by the reagents (e.g. neurotoxicity). In this article, however, we will not focus on this issue because an allogeneic setting will not be able to solve this issue.

One point to be addressed here is that autologous TCR-T cell therapy is not necessarily safe in terms of the risk of attacking the recipient, i.e. graft vs. host disease (GVHD). This may take place because transferred TCR proteins can form various new TCRs by mispairing with endogenous TCR proteins. To avoid this risk, methods have been developed where expression of the endogenous TCR gene is suppressed by using small interfering RNA (siRNA) (1, 2).

To address these issues, strategies using allogeneic T cells are being developed. One of them is a method in which T cells are collected from healthy donors and used as the starting material (Fig. 2, middle lane). In this case, since the material is collected from a healthy donor, it can be prepared as cryopreserved cell stocks in advance and thus can be used as an off-the-shelf (immediate delivery type) cell preparation. In addition, a certain level of quality can be guaranteed by pre-testing.

On the other hand, allogeneic T cells have the problem of being rejected by the recipient’s immune system. In this regard, techniques such as genome editing are being used to delete human leukocyte antigen (HLA) genes in order to avoid immune rejection by recipient T cells (3, 4). The effect of HLA depletion in cell therapy and the immune rejection by NK cells instead of T cells will be discussed in detail later.

Apart from the rejection issue, allogeneic T cells have another serious issue: the risk of GVHD. In general, a few percentage of peripheral T cells can be potentially activated by HLA-mismatched donor cells. While such T cells, which are called “allo-reactive” T cells, are known to play major roles in graft rejection in regular transplantation cases, they may cause GVHD when allogeneic T cells are transferred to the patient. To address this issue, several methods have been developed, where TCR gene is simply knocked out (5, 6), or disrupted by inserting a CAR gene into the TCR gene locus (7), using genome-editing technology.

However, even if the use of allogeneic T cells taken from another person can be safely conducted, problems still remain. The number of cells that can be obtained is limited, and it is difficult to reduce the cost. As aforementioned, quality can be tested in advance, but if multiple steps of genetic modification are conducted, variations will occur, making it difficult to produce homogeneous cell preparations.

Here we argue that these problems can be solved by using methods in which T cells are produced from pluripotent stem cells such as ES (embryonic stem) cells or iPS (induced pluripotent stem) cells that have been transduced with TCR or CAR genes beforehand (Fig. 2, bottom lane). Since T cells can be expanded by more than 10^4^-fold after reaching the mature stage without getting exhausted (8), it will be possible to obtain the necessary number of cells by establishing a mass-production system. Another merit is that T cells can be cryopreserved until just before use, while in general other types of differentiated cells/tissues are difficult to be cryopreserved. The usage of mass-production system and cryopreservation will make it possible to lower production costs for commercialization. In addition, the problem of rejection can be avoided by using iPS cells with certain HLA types. The use of iPS cells carrying HLA types that are less likely to be rejected will be explained in the next section.

**Figure 3.**
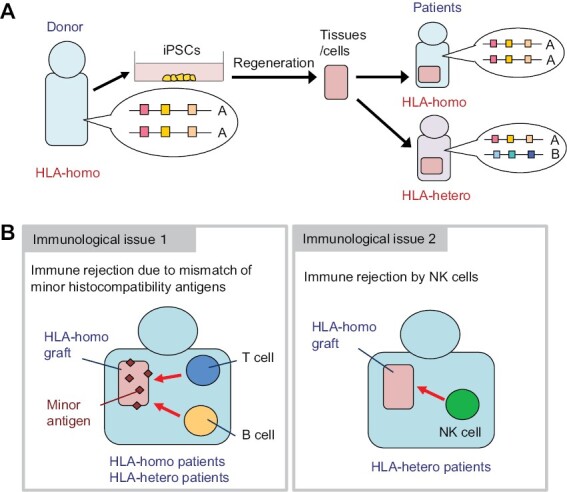
The iPS Cell Stock Project and possible immune reactions occurring in this setting. (A) The outline of the iPS Cell Stock Project. When iPS cells are generated from a person who has the same HLA haplotype in both alleles in a homozygous manner (HLA-homo), tissue regenerated from such iPS cells is less likely to be rejected when transplanted to a person with the same HLA-homo genotype and to a person who has the same HLA haplotype on one allele (HLA-hetero). Applying this principle, the CiRA-F (Kyoto University) has established and distributed iPS cell stocks for the use in allogeneic transplantation settings. Currently, the iPS cell lines with the top four most frequent HLA-homo haplotypes are distributed, covering approximately one-third of the Japanese population. (B) Immunological issues in homo–homo and homo–hetero transplantation. Issue 1: the mismatch of minor histocompatibility antigens. Minor histocompatibility antigens expressed in grafts do not match the recipients, except in the case of monozygotic twins. Immune rejection may take place when immune cells recognize these antigens in both homo–homo and homo–hetero transplantation cases. Issue 2: NK cell-mediated immune reactions. NK cells in the HLA-hetero recipient may recognize the regenerated HLA-homo graft and exert cytotoxic activity. Thus, this reaction occurs only in homo–hetero transplantation cases.

We are currently developing a method using the abovementioned iPS cell-derived T cells for clinical trials. Specifically, we are preparing for an investigator-initiated clinical trial targeting acute myeloid leukemia that expresses the WT-1 antigen, in collaboration with the Department of Hematology and Oncology at Kyoto University Hospital, the Center for Research and Application of Cellular Therapy (C-RACT), and the Institute for Advancement of Clinical and Translational Science (iACT). We are going to use the TCR gene that has already been shown to be effective and safe in clinical trials (9), and as the iPS cells, the iPS cell line that is homozygous for the most frequent HLA haplotype in the Japanese population, provided by the iPS Cell Stock Project conducted by the Center for iPS Cell Research and Application Foundation (CiRA-F) (10), is used (Fig. 3A). We have shown that CTLs produced by this method exert the effect of suppressing tumor growth in a patient-derived xenograft model of renal cell carcinoma (11). At present, our group is producing T cells from iPS cells in the cell processing center of Kyoto University Hospital, under the control of C-RACT.

## The principle of the iPS Cell Stock Project

ES cells are produced by culturing the inner cell mass of blastocysts, and human ES cells were established in 1998. ES cells have been expected to be applicable as material cells in regenerative medicine, but it is difficult to match the HLA type of ES cells to individual patients. Therefore, the immunological problem of graft rejection was inevitable. In 2006, Takahashi and Yamanaka developed the iPSC technology using mouse cells (12), followed by the establishment of human iPS cells (13). Since iPS cells can be produced from somatic cells of the patient, they were expected to be able to solve the immunological problems.

However, it soon became clear that it would take too much time and cost to actually generate iPS cells for each patient, and so regenerative medicine using iPS cells has been promoted on the expectation that it will be conducted in allogeneic transplantation settings. In 2013, CiRA started the iPS Cell Stock Project to create and distribute iPS cells for allotransplantation. In this project, iPS cells have been generated using cells harvested from healthy adult volunteers or umbilical cord blood with homozygous HLA haplotypes (HLA-homo). It is expected that T cells in recipients will not attack the graft not only when transplanted to a person who is also HLA-homo (homo–homo transplantation), but also when transplanted to a person who has a heterozygous HLA haplotype (HLA-hetero) (homo–hetero transplantation) (14–16) (Fig. 3A). From the recipient’s T cells’ point of view, it is difficult for them to recognize the HLA as a foreign substance, because the recipient also has the same HLA.

For example, since the most frequent HLA haplotype in Japan is possessed by approximately 17% of the Japanese population, it is calculated that the tissues/cells regenerated from iPS cells with the most frequent HLA haplotype can be transplanted in one out of six people. The top 70 types of iPS cells can cover approximately 70% of the Japanese population (15).

## Immunological issues remaining after HLA matching

It is not the case that the strategy of using HLA-homo iPS cells has solved the immunological problems. As mentioned above, in principle, a T-cell response to the mismatched HLA does not occur in either homo–homo or homo–hetero transplantation. However, there are still some remaining problems. They are (i) rejection due to minor histocompatibility antigen (hereinafter referred to as minor antigen) mismatch and (ii) rejection by NK cells (Fig. 3B).

Minor antigens are proteins with different amino acid sequences caused by genetic polymorphisms. When these are targeted, an immune response by T cells and antibodies eventually occurs. Except for identical twins as donors, this immune response occurs even in HLA-matched transplants. Therefore, in both homo–homo and homo–hetero transplants, immune responses to minor antigens occur, and unless immunosuppressants are used, the grafts are likely to be eventually rejected. On the other hand, responses by NK cells can occur only in the case of homo–hetero transplantation. It is generally difficult to predict immune responses to minor antigens, but responses by NK cells can be predicted. Hereafter, we introduce our research on the immune response of NK cells taking place in homo–hetero transplantation.

## Inhibitory receptors of NK cells and their ligands

**Figure 4.**
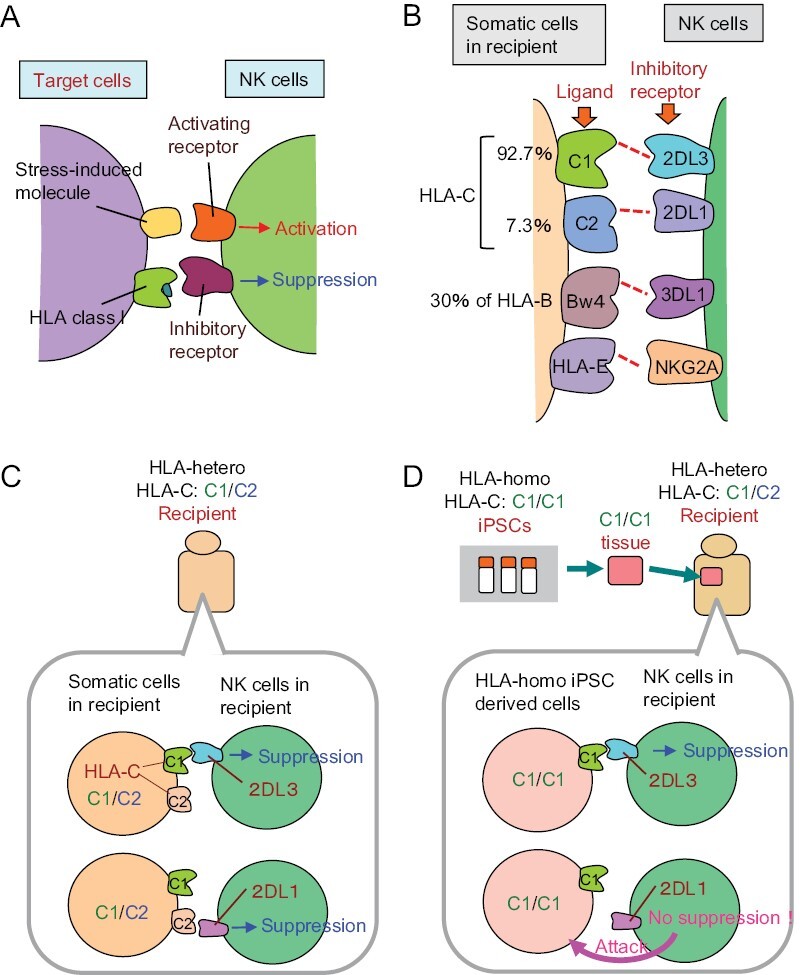
The mechanism of NK cell-based immune responses. (A) NK cells sense molecules expressed on cells under stress, and attack them. On the other hand, inhibitory receptors of NK cells sense HLA class I molecules and generate inhibitory signals, serving as a safeguard system. (B) Inhibitory receptors and ligands of human NK cells. HLA-C molecules function as the major ligands of inhibitory receptors expressed on NK cells. HLA-C molecules can be classified into two types: C1 and C2. The receptors for C1 and C2 are KIR2DL3 and KIR2DL1, respectively. Some HLA-B epitopes (~30%), namely HLA-Bw4 ligand, serve as a ligand for KIR3DL1. HLA-E acts as a ligand for NKG2A. (C) NK cells in recipients carrying HLA-C1/C2 are inhibited by the ligand of either HLA-C1 or -C2 expressed on somatic cells. (D) It is predicted that HLA-hetero C1/C2 recipient NK cells may sense the absence of the C2 molecule on the graft when the graft derived from HLA-homo iPSCs carrying HLA-C1/C1 is transplanted, resulting the killing of the graft.

NK cells sense molecules expressed by cells under some stress and attack the cells. On the other hand, NK cells do not attack cells expressing HLA class I, serving as a safeguard system (Fig. 4A). In other words, NK cells sense the loss of class I on cells and attack them. This mechanism works as a backup system for CTLs; NK cells kill infected and cancer cells that have escaped from CTLs by terminating class I expression.

In humans, there are three “classical,” highly polymorphic types of HLA class I: HLA-A, HLA-B, and HLA-C. HLA-C, some HLA-B epitopes, and the “non-classical” non-polymorphic HLA-E act as the main ligands for the inhibitory receptors of NK cells (17) (Fig. 4B). HLA-C can be classified into two groups, namely C1 and C2, and the inhibitory receptors for C1 and C2 types are called KIR (killer cell immunoglobulin-like receptor) 2DL3 (hereafter referred to as 2DL3) and KIR2DL1 (2DL1), respectively. Some HLA-B epitopes (i.e. HLA-Bw4; about 30% of HLA-B) act as ligands for KIR3DL1 (3DL1). HLA-E acts as a ligand for NKG2A, an inhibitory receptor belonging to the C-type lectin family.

## Immune reactions by NK cells that can occur during homo–hetero transplantation

Now consider NK cells in the recipient body having HLA-C of both C1 and C2 types (HLA-C1/C2) (Fig. 4C). Since the inhibitory receptors of NK cells are randomly expressed on a cell-by-cell basis, there exist NK cells expressing only 2DL3, and NK cells expressing only 2DL1. These NK cells are suppressed by HLA-C1 or HLA-C2 molecules, respectively. When tissues/cells regenerated from HLA-homo iPS cells with HLA-C1/C1 are transplanted, the regenerated tissues/cells, which do not have HLA-C2 molecules, cannot suppress 2DL1-expressing NK cells in the recipient body (Fig. 4D). Accordingly, it was assumed that they were killed by activated NK cells.

**Figure 5.**
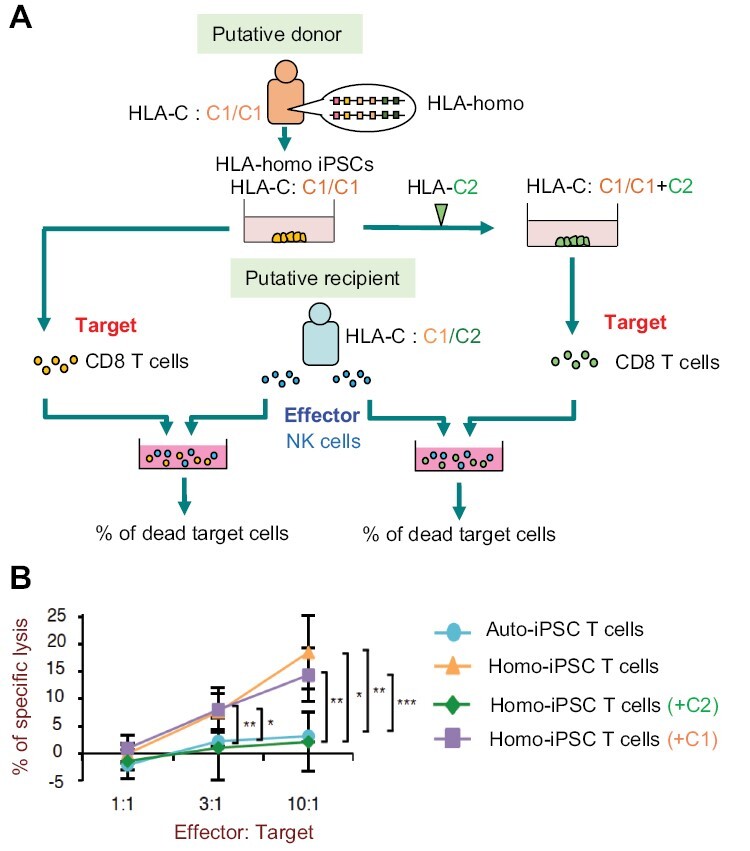
Cytotoxic activity mediated by NK cells in the homo–hetero transplantation setting. (A) Schematic illustration of the experimental design. iPS cells were established from an HLA-homo individual as a putative donor. The HLA-homo-C1/C1 iPS cells were further transduced, using a lentiviral system, with HLA-C2 that is identical to the putative recipient. As target cells, T cells were differentiated from these iPS cells. As effector cells, NK cells were collected from peripheral blood mononuclear cells of a putative recipient, who was an HLA-hetero person carrying both HLA-C1 and -C2. As a negative control, HLA-homo-C1/C1 iPS cells transduced with the HLA-C1 gene were also produced. After effector and target cells were co-cultured for 6 h in different effector: target ratios, the percentage of dead target cells was measured by the Cr^51^-release assay. (B) Cytotoxicity assay of NK cells isolated from the putative recipient against target T cells. *P < .05, **P < .01, ***P < .001; Student’s *t*-test.

Therefore, we first investigated whether HLA-hetero NK cells elicit an immune response against HLA-homo iPS cell-derived cells (18). We generated iPS cells from a healthy volunteer who was HLA-homo, and regenerated T cells as target cells from such HLA-homo iPS cells (Fig. 5A, left lane). NK cells were collected from HLA-C1/C2 type healthy volunteers and used as effector cells. Autologous T cells regenerated from the recipient-derived iPS cells were not killed at all (Fig. 5B, line indicated by circular dots). On the other hand, the rate of dead cells in allogeneic T cells was significantly increased (Fig. 5B, line indicated by triangular dots). Thus, the NK cells were shown to attack HLA-homo regenerated cells.

Next, we investigated a method to avoid the immune response by NK cells. We over-expressed the HLA-C2 molecules that were possessed by recipients in HLA-homo donor iPS cells (Fig. 5A, right lane). As expected, regenerated allogeneic T cells that expressed exogenous HLA-C2 molecules were not killed (Fig. 5B, line indicated by diamond-shaped dots). These results indicate that the rejection by NK cells can be avoided by over-expressing, the same HLA-C2 molecule as possessed by the recipient, in the regenerated tissue.

## The current status of, and issues facing, initiatives toward genuine universal cells

As mentioned in the previous section, problems remain even if the HLA homo–hetero transplantation system is used. Also, it is not realistic for business companies to prepare a large number of HLA-homo iPS cell lines. Medical professionals have wondered if they could somehow prepare genuine “universal-use” material cells.

**Figure 6.**
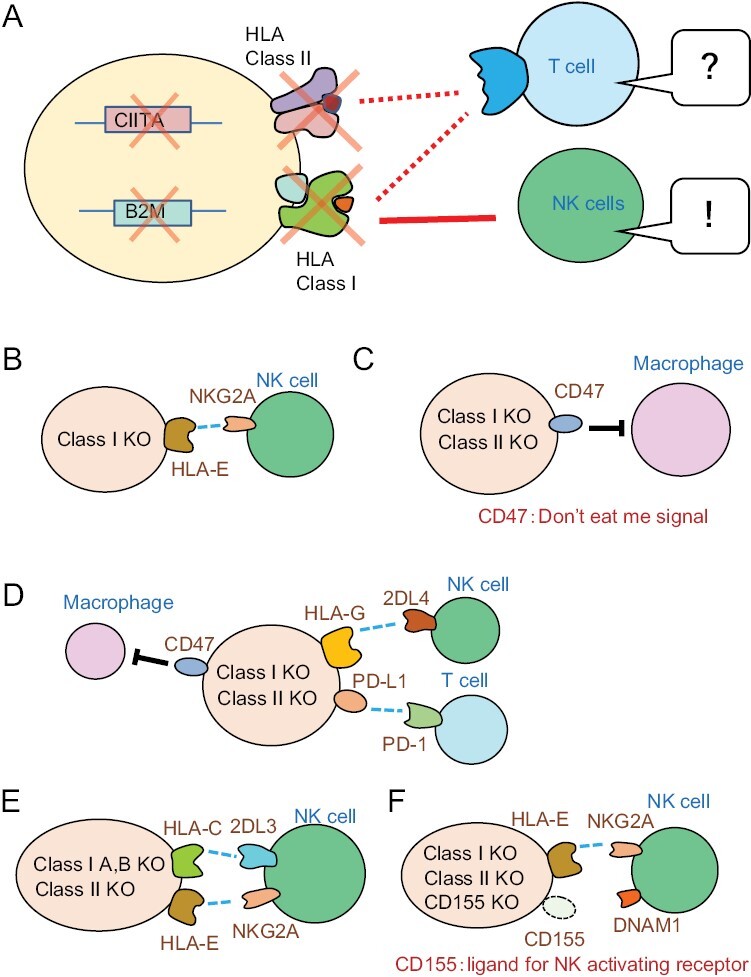
Attempts to create universal cells that are free from attack by T cells and NK cells. (A) Knockout of B2M (beta-2-microglobulin) and CIITA results in the inability to express HLA class I and class II, respectively. This operation renders T cells unresponsive, whereas NK cells sense the loss of class I and begin to attack the cells. (B) Forced expression of HLA-E to avoid rejection by a portion of NK cells. (C) Forced expression of CD47 to avoid phagocytosis by macrophages. (D) Forced expression of HLA-G to avoid rejection by a portion of NK cells, CD47 to avoid phagocytosis by macrophages, and PD-L1 to avoid attack by T cells. (E) Leave one allele of HLA-C to retain an antigen-presenting ability for T cells and to avoid rejection by NK cells. HLA-E expression is retained to avoid rejection by a portion of NK cells. (F) Knockout of CD155, which is a ligand for the activating receptor DNAM1 on NK cells, and addition of forced expression of HLA-E to avoid rejection by a portion of NK cells.

Now, knowing that the rejection by the patient’s immune cells is primarily due to the mismatch of HLA between donor and recipient, it is natural to think that the HLA should be deleted in material cells. In order to eliminate HLA expression, a method of knockout (KO) of the B2M gene in the case of class I, and KO of the CIITA gene, involved in transcriptional regulation of class II, is usually taken (Fig. 6A). Since NK cells are suppressed by inhibitory receptors that recognize class I molecules as ligands, class I-deficient cells may become targets of NK cells.

Methods to avoid the rejection by NK cells have been reported by multiple groups as follows:

- A method for forced expression of HLA-E in pluripotent stem cells (19) (Fig. 6B). NKG2A on NK cells senses HLA-E over-expressed on regenerated cells and conducts an inhibitory signal. However, since only 30%–50% of NK cells express NKG2A, this method is not effective against NKG2A-negative NK cells.
- HLA-deficient cells expressing CD47, one of the “don’t eat-me signals”, to protect grafts from macrophage attack (20) (Fig. 6C). However, it is considered that this method can suppress phagocytic cells but cannot effectively avoid the rejection by NK cells.
- A method for forced expression of CD47, PD-L1, and HLA-G in HLA-deficient cells (21) (Fig. 6D). PD-L1 suppresses activated T cells. HLA-G is expressed in placental trophoblasts during pregnancy and is believed to act on maternal NK cells to protect the fetus (22), but there is also a report that HLA-G rather activates NK cells (23); thus the effect remains unclear.
- A method to KO the CIITA gene for class II, and to KO HLA-A and B for class I, leaving only one HLA-C (24) (Fig. 6E). It is said that the CiRA-F will focus on this series from now on, and that 95% of Japanese people can be covered by preparing the top seven types of HLA-C. By leaving HLA-C, the ability to present antigens to T cells is preserved to some extent. On the other hand, by leaving HLA-C and HLA-E, they will avoid the attack by NK cells. However, because of the lack of the HLA-Bw4 ligand (30% of HLA-B), KIR-ligand mismatch will occur in Bw4-positive recipients when Bw4-negative tissues/cells are transplanted (the frequency of this is expected to be about 50%) (25).
- A method of CD155 KO in donor cells (CD155 is a ligand of the NK cell-activating receptor DNAM1) (26) (Fig. 6F). The effect may be limited, since not all NK cells express DNAM1, and the graft may express multiple other types of activating ligands.

In summary, various methods have been explored, but no method seems to be very effective. Further research and development are necessary to completely avoid attack by NK cells.

## Conclusion

It is believed that cell medicine will continue to develop significantly, centered on T cells. In the process, it is believed that the leading role will surely change from autologous T cells to allogeneic T cells.

As mentioned in this article, the strategy of using pluripotent stem cells as a material is very promising as a method for generating allogeneic T cells, but some issues still remain. In order to increase versatility, it is necessary to delete the HLA, but in that case, there is a problem that rejection by NK cells is induced. Many groups have taken various approaches to address these issues, but no one method seems to be decisive yet. Since the production of allogeneic universal cells is a very important issue for cell medicine and regenerative medicine, it is necessary to strongly promote research and development toward a solution.

Recently, it has become possible to produce high-quality iPS cells for each patient, and CiRA-F has been preparing such an approach as the “my iPS cells” project. In this context, the use of iPS cell method in autologous setting, which should be free from rejection issue, may also remain a viable method in the future.
